# 4-[(5-Methyl-1H-pyrazol-3-yl)amino]-2H-phenyl-1-phthalazinone Inhibits MCPyV T Antigen Expression in Merkel Cell Carcinoma Independent of Aurora Kinase A

**DOI:** 10.3390/cancers15092542

**Published:** 2023-04-28

**Authors:** Roland Houben, Pamela Alimova, Bhavishya Sarma, Sonja Hesbacher, Carolin Schulte, Eva-Maria Sarosi, Christian Adam, Thibault Kervarrec, David Schrama

**Affiliations:** 1 Department of Dermatology, Venereology und Allergology, University Hospital Würzburg, 97080 Würzburg, Germany; 2 Department of Pathology, Centre Hospitalier Universitaire de Tours, INRA UMR 1282 BIP, 37200 Tours, France

**Keywords:** Merkel cell carcinoma, polyomavirus, large T antigen, phthalazinone pyrazole, glycogen synthase kinase 3, GSK3

## Abstract

**Simple Summary:**

Treatment of Merkel cell carcinoma—a deadly skin cancer frequently caused by the Merkel cell polyomavirus—still poses challenges. Since the tumor cells depend on expression of viral oncogenes—named T antigens—targeting the expression of these T antigens appears as a potential therapeutic strategy. Therefore, we screened a kinase inhibitor library for compounds repressing growth of Merkel cell carcinoma cells specifically by inhibiting expression of these viral proteins and identified a compound previously described as an inhibitor of Aurora kinase A (AURKA). However, we provide evidence that the T-antigen repressing effect is not related to inhibition of AURKA but probably to a hitherto unknown GSK3-inhibitory activity of the compound, whose potential use a therapeutic is demonstrated in immunocompromised mice transplanted with human MCC.

**Abstract:**

Merkel cell carcinoma (MCC) is frequently caused by the Merkel cell polyomavirus (MCPyV), and MCPyV-positive tumor cells depend on expression of the virus-encoded T antigens (TA). Here, we identify 4-[(5-methyl-1H-pyrazol-3-yl)amino]-2H-phenyl-1-phthalazinone (PHT)—a reported inhibitor of Aurora kinase A—as a compound inhibiting growth of MCC cells by repressing noncoding control region (NCCR)-controlled TA transcription. Surprisingly, we find that TA repression is not caused by inhibition of Aurora kinase A. However, we demonstrate that β-catenin—a transcription factor repressed by active glycogen synthase kinase 3 (GSK3)—is activated by PHT, suggesting that PHT bears a hitherto unreported inhibitory activity against GSK3, a kinase known to function in promoting TA transcription. Indeed, applying an in vitro kinase assay, we demonstrate that PHT directly targets GSK3. Finally, we demonstrate that PHT exhibits in vivo antitumor activity in an MCC xenograft mouse model, suggesting a potential use in future therapeutic settings for MCC.

## 1. Introduction

Merkel cell carcinoma (MCC) is an aggressive neuroendocrine skin cancer with increasing incidence that primarily affects the elderly [1,2]. Two subtypes can be distinguished; one is characterized by high mutational burden with UV signature, while the key feature of the second, more frequent MCC subtype is the presence of an integrated Merkel cell polyomavirus (MCPyV) genome [3,4]. For MCPyV-positive MCC, there is mounting evidence for a causal link between MCPyV and tumorigenesis [5], including a recent mouse model in which expression of MCPyV-encoded oncogenes (small and Large T antigen (sT and LT)) could induce tumors almost perfectly resembling human MCC [6]. With respect to therapeutical implications, even more important than the role in tumorigenesis is the fact that established MCPyV-positive MCC cells are dependent on expression of the MCPyV T antigens (TA) [7,8].

Interestingly, while sT is the wild type, the LT proteins expressed in MCC are characteristically truncated (either due to stop codon mutations or as a result of integration) [9,10], suggesting that functional domains located in the C-terminus are pernicious for tumor cell growth. In contrast, since the RB binding domain is always intact, RB1 inactivation by LT seems essential for MCC development [9,11]. This notion has recently been nicely confirmed in a mouse model in which MCPyV-TA-induced tumorigenesis was abolished when wild-type RB1 was replaced by an RB1 protein incapable of binding LT [12]. While RB1 appears to be the LT target with outstanding importance in MCC [13], MCPyV small T antigen (sT) has been demonstrated to have impact on several oncogenic pathways possibly involved in MCC tumorigenesis [14]. Among those interactions are (i) targeting of the transcription factor MYCL to EP400 complex [15] and thereby indirectly repressing p53 [16] and HLA-I expression [17], (ii) altering translation via 4E-BP1 [7], (iii) activation of non-canonical NF-kB signaling [18] and (iv) inhibition of the protein ubiquitinase FBW7 and thereby stabilizing other oncoproteins (e.g., LT) [19]. Although these crucial functions of the MCPyV-TAs in MCPyV-positive MCC have been revealed, so far no therapeutic approach targeting the MCPyV-TAs has entered the clinic.

Until 2016, there were no therapeutical options providing a significant survival benefit for patients with advanced MCC [20], for whom a 5-year survival rate of only 18% had been determined [21]. Indeed, although tumors responded to radio- and chemotherapy, those therapies had no impact on overall survival [22]. However, with the advent of the PD-1/PD-L1 checkpoint inhibitors, the situation has been improved significantly [23,24]. Approximately half of the treated patients are responding [25,26], and strikingly, the responses are frequently durable, with a reported ongoing response rate among the responders of 73% after 3 years [27]. However, approximately 50% of the treated MCCs are still primarily resistant to the approved PD-1/PD-L1 inhibitors, and therefore, there is still a need for alternative therapeutic approaches. For MCPyV-positive MCC, targeting expression of the T antigens, which are essential for the tumor cells, appears as a possible strategy.

To this end, we applied a functional screen to search for compounds which might affect growth of MCPyV-positive MCC cells specifically through repressing T-antigen expression. One such compound was 4-[(5-methyl-1H-pyrazol-3-yl)amino]-2H-phenyl-1-phthalazinone (PHT), a reported inhibitor of Aurora kinase A [28]. Here, we demonstrate that inhibition of TA expression in MCC cells by PHT is not linked to inhibition of Aurora kinase A. Furthermore, repression of MCC xenograft growth in mice suggests a potential therapeutical usefulness of PHT.

## 2. Material and Methods

### 2.1. Ethics Statement

With the approval of the Regierung von Unterfranken (RUF 55.2.2-22532.2-925-18), animal experiments were conducted according to the EU Directive 2010/63/EU for animal experiments.

### 2.2. Cell Culture

The MCPyV-positive MCC cell lines analyzed in this study were PeTa (kind gift of Jochen Utikal, Mannheim, Germany) [29], WaGa, BroLi (both established in our lab), MKL-2 (all described in [8]) and MKL-1 (both kind gifts of Patrick Moore, Pennsylvania) [30]. For lentivirus production, we additionally used HEK-293T cells. All cell lines were grown in RPMI 1640 supplemented with 10% FCS, 100 U/mL penicillin and 0.1 mg/mL streptomycin.

### 2.3. Vectors and Lentiviral Infection

For inducible knockdown of AURKA, we used a previously described lentiviral single-vector system (gene bank accession number MH749464), allowing Doxycycline-inducible shRNA expression [31]. Annealed oligonucleotides were cloned into the vector induc-shRNA EYFP P2A puro, resulting in vectors encoding for shRNAs targeting the following *AURKA* sequences: *AURKA* shRNA1 GCA CCA CTT GGA ACA GTT TAT and *AURKA* shRNA2 GCC AAT GCT CAG AGA AGT ACT. The pIH vector coding for constitutive expression of TA with truncated LT has been described before [32]. For assessment of the transcriptional activity of the MCPyV noncoding control region (NCCR) or β-catenin activity, we used our previously described NCCR or β-catenin reporter, respectively [31,33].

Lentiviral particles for transduction of cells were generated with HEK293T cells as previously described [33].

### 2.4. Screen for Compounds Repressing NCCR-Dependent Transcription

The assay to identify kinase inhibitors affecting growth of MCC cells specifically through repression of MCPyV-TA expression has been described in detail elsewhere [33]. Growth differences between MCPyV-positive MKL-1 cells and MKL-1 cells expressing additionally ectopic MCPyV-TA were measured by flow cytometry. To this end, the MKL-1 cells transduced with pIH MCPyV-TA encoding for sT and a truncated LT with a protein size of 278 amino acids were additionally transduced with a vector encoding for green fluorescent protein (GFP). A total of 180 different kinase inhibitors (10 μM) included in a library purchased by Cayman Chemicals (10505) were applied to mixtures containing 80% parental MKL-1 cells. After seven days, flow cytometry (Cytoflex; Beckman Coulter, Krefeld, Germany) was used to determine changes in the proportion of green fluorescent cells.

### 2.5. Immunoblotting

Immunoblotting was performed as previously described [32]. The following antibodies were used in this study: anti-MCPyV-LT (CM2B4; Santa Cruz Biotechnology, Dallas, TX, USA), anti-MCPyV-TA (2T2 hybridoma supernatant; [34]), anti-AURKA (1G4; Cell Signaling, Danvers, MA, USA), anti-β-tubulin (TUB 2.1; Sigma-Aldrich, Taufkirchen, Germany) and anti-vinculin (V9131; Sigma Aldrich).

### 2.6. Real-Time PCR

Real-time PCR with primers for MCPyV-sT and -LT as well as RPLP0 were performed as described previously [10].

### 2.7. EdU Incorporation Assay

The assay has been described in more detail elsewhere [33]. Briefly, cells were incubated for 30 min at 37 °C with a culture medium supplemented with 10 µM EdU, followed by fixation in 4% formaldehyde in PBS and permeabilization in 90% ice-cold methanol. Finally, cells were re-suspended in PBS with 0.1% Triton X-100, 10 mg/mL RNAse A and Hoechst 33342 and analyzed by flow cytometry on a Cytoflex (Beckman Coulter).

### 2.8. Animal Experiments

A total of 5 × 10^6^ MKL-1 suspended in 100 μL medium consisting of 50% phosphate buffered saline (PBS) and 50% Matrigel (Corning, Wiesbaden, Germany) were injected subcutaneously in five-week-old female NOD.CB17/Prkdc^scid^ mice (Charles River, Sulzfeld, Germany). Tumor volume was calculated using daily measurements with a vernier caliper and the formula V = π/6 × a^2^ × b (a: length; b: height). Randomization was performed when the volume reached about 150 mm^3^. The control group consisted of 5 mice, since no tumor was observed in one mouse, and the treatment group consisted of 6 mice. The animals in the treatment group were subjected to daily intraperitoneal injections with 30 mg/kg PHT dissolved in 200 µL 2% DMSO in PBS. The animals in the control group received the same volume of 2% DMSO in PBS. Once the tumors in the control group reached the maximum tolerable size, the experiment was terminated.

### 2.9. GSK3 Kinase Assay

The GSK3-inhibitory effect of PHT was evaluated using a commercial GSK3 kinase assay (Promega, Walldorf, Germany) in combination with an assay quantifying ADP produced in the kinase reaction by luciferase (ADP-GloTM Kinase Assay; Promega). We followed the instructions of the manufacturer for determining dose—response curves by titrating the different drugs (CHIR99021, PHT or alisertib) to the GSK3 kinase reaction. Light produced by the luciferase was measured in a Tecan multi-well plate reader.

### 2.10. Phospho-Histone H3 Flow Cytometry

After the indicated treatments for 24 h, cells were fixed in 4% formaldehyde in PBS and permeabilized in 90% ice-cold methanol. After incubation with an alexa fluor 488 labeled anti-phospho-histone H3 antibody (D2C8; Cell Signaling) for 30 min, cells were analyzed by flow cytometry on a Cytoflex (Beckman Coulter).

### 2.11. Statistics

Statistical tests were performed using Prism 9.1.1 (GraphPad Software). For statistical analysis of more than two groups, we used paired one-way ANOVA with the Tukey multiple comparison test. One sample *t*-test was performed to analyze deviation to a reference group. An unpaired *t*-test was used to compare the values obtained for the area under the curve of the tumor sizes (baseline set to zero and normalized to the size at the start of treatment).

## 3. Results

### 3.1. Screening for Kinase Inhibitors Targeting TA Expression

**Figure 1:**
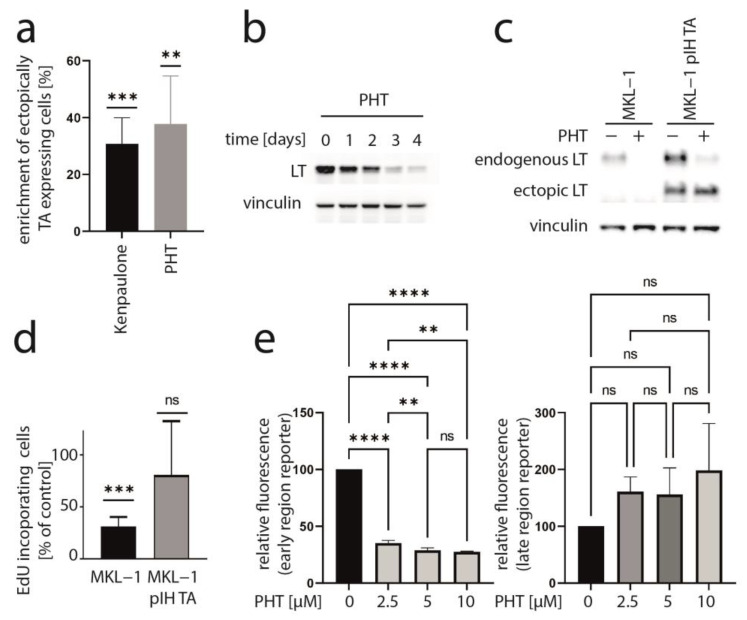
PHT inhibits growth of MKL-1 cells by repressing TA expression. (**a**) A mixed cell culture of MCPyV-positive MKL-1 cells and MKL-1 cells additionally expressing ectopic MCPyV TA (MKL1 pIH TA) as well as GFP were treated with different kinase inhibitors (10 μM). Among the compounds inducing enrichment of the GFP-positive cells were six GSK3 inhibitors [33] and PHT. Mean enrichment (±SD) of at least five independent experiments is shown for Kenpaulone, the most efficient GSK3 inhibitor and PHT. (**b**) Time course of LT expression in MKL-1 cells treated with 10 µM PHT followed by immunoblot analysis of lysates harvested at the indicated time points. (**c**,**d**) MKL-1 and MKL1 pIH TA cells were treated with 10 µM PHT for four days. MCPyV-LT expression was analyzed by immunoblot (**c**) and proliferation by performing the EdU incorporation assay (**d**). EdU-positive cells were recorded and mean values (+SD) relative to untreated controls from three independent experiments are depicted. (**e**) MKL-1 cells infected with an NCCR reporter construct in which the expression of mNeongreen represents that of T antigens while mRuby3 represents the expression of the late region were treated with the indicated concentrations of PHT. Mean values (±SD) of the mean fluorescence intensities (normalized to controls) of four independent experiments are depicted. Significance levels: ns: not significant; **: ≤0.01; ***: ≤0.001; ****: ≤0.0001.

We recently presented data of a kinase inhibitor library screen aiming to find inhibitors which might impair MCC cell growth by suppressing T-antigen expression [33]. In this regard, we utilized a mixed cell culture assay for which parental MCPyV-positive MKL-1 cells were endogenously expressing MCPyV-T antigen with MKL-1 cells additionally expressing ectopic TA (pIH TA) and GFP. Applying drugs from a library with 180 different kinase inhibitors, those substances were revealed which led to an enrichment of ectopically TA-expressing cells, suggesting that they might specifically repress endogenous NCCR-regulated TA expression. As recently reported, six of the compounds which induced a more than 20% relative increase in MKL-1 pIH TA cells following a 7-day treatment were GSK3 inhibitors [33]. An enrichment of ectopically TA-expressing cells comparable to the most efficient GSK3 inhibitor Kenpaulone was also observed for a phthalazinone pyrazole (specifically 4-[(5-methyl-1H-pyrazol-3-yl)amino]-2H-phenyl-1-phthalazinone [PHT]) (Figure 1a), which has been reported to be a specific inhibitor of Aurora kinase A [28]. PHT corresponds to compound #16 in the original publication [28].

### 3.2. PHT Represses MCC Growth by Repressing MCPyV-TA Expression

**Figure 2:**
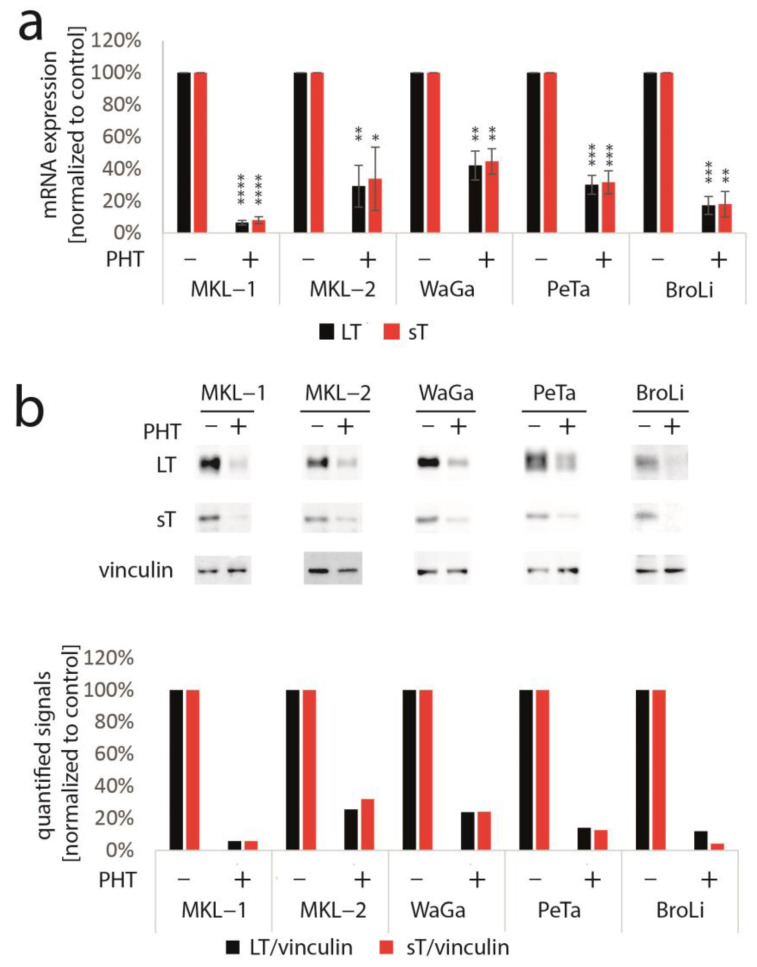
PHT represses TA mRNA and protein expression in five different MCC cell lines. The various cell lines were treated with PHT (10 µM) for three (mRNA analysis) or five days (protein analysis). (**a**) TA mRNA expression was analyzed using real-time PCR and specific primers for *MCPyV-LT* and *sT*. *RPLP0* served as endogenous control for normalization. Given are mean values (±SD) of five independent experiments. (**b**) LT and sT expression were examined using immunoblot. In the bar graph, quantified signals of the above immunoblot are displayed. Significance levels: *: ≤0.05; **: ≤0.01; ***: ≤0.001; ****: ≤0.0001.

To determine whether TA expression is repressed by PHT, we performed immunoblot analysis with lysates derived from PHT-treated MKL-1 cells. Indeed, MCPyV-LT expression levels decreased over time (Figure 1b). Analyzing LT expression of MKL-1 and MKL-1 cells additionally expressing ectopic MCPyV-TA revealed that—as predicted by the mixed cell culture assay—only endogenous LT was repressed while expression of ectopic LT controlled by a LT3 promoter was unaffected (Figure 1c). To determine the effect of PHT on proliferation, we next performed the EdU assay on day 4 when TA expression is clearly reduced. This assay revealed that PHT treatment impaired DNA synthesis in parental MKL-1 cells, while in cells additionally expressing ectopic MCPyV-TA, this DNA synthesis inhibition triggered by PHT was much weaker (Figure 1d), implying that PHT by inhibiting NCCR-controlled TA expression, cell cycle progression in MKL-1 is affected. In contrast, survival was not affected by 72 h of PHT treatment, neither in MKL-1 cells nor in fibroblasts (Supplementary Figure S1). To confirm a specific effect of PHT on the viral MCPyV promoter, we applied a reporter gene assay using a lentiviral vector in which mNeonGreen and mRuby3 are expressed under the control of the MCPyV-NCCR, representing early and late region genes, respectively [31]. Indeed, we found a highly significant reduction of green fluorescence in NCCR reporter transduced MKL-1 cells upon PHT treatment, suggesting repression of MCPyV early gene transcription, while late gene transcription showed a slight, but statistically not significant, increase (Figure 1e). To confirm this notion and to evaluate whether repression of early gene expression is restricted to MKL-1 cells or a general feature of MCC cells, we next treated five different MCC cell lines with PHT and analyzed sT and LT mRNA levels using real-time PCR, and LT and sT protein levels with immunoblot. Indeed, PHT-induced suppression of sT as well as LT was evident on mRNA (Figure 2a) and protein level (Figure 2b) in all five cell lines.

### 3.3. Aurora Kinase A Is Not the Crucial Target of PHT Mediating TA Repression

**Figure 3:**
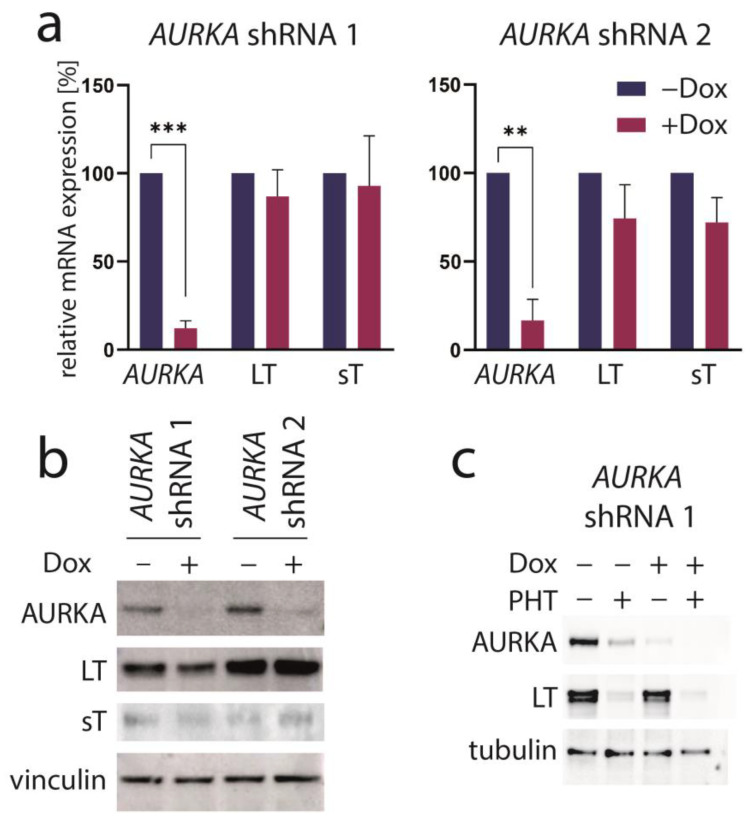
AURKA knockdown does not repress TA expression. MKL-1 cells were transduced with vectors allowing Dox-inducible expression of two different shRNAs targeting *AURKA*. After five days of Dox treatment, (**a**) *AURKA* as well as MCPyV sT and LT mRNA expression were analyzed using real-time PCR with RPLP0 serving as internal standard. Mean values (+SD) normalized to control for three independent experiments are depicted. (**b**) Immunoblot of total cell lysates harvested after five days of Dox treatment. (**c**) Immunoblot following combined treatment with Dox and PHT. Significance levels: **: ≤0.01; ***: ≤0.001.

**Figure 4:**
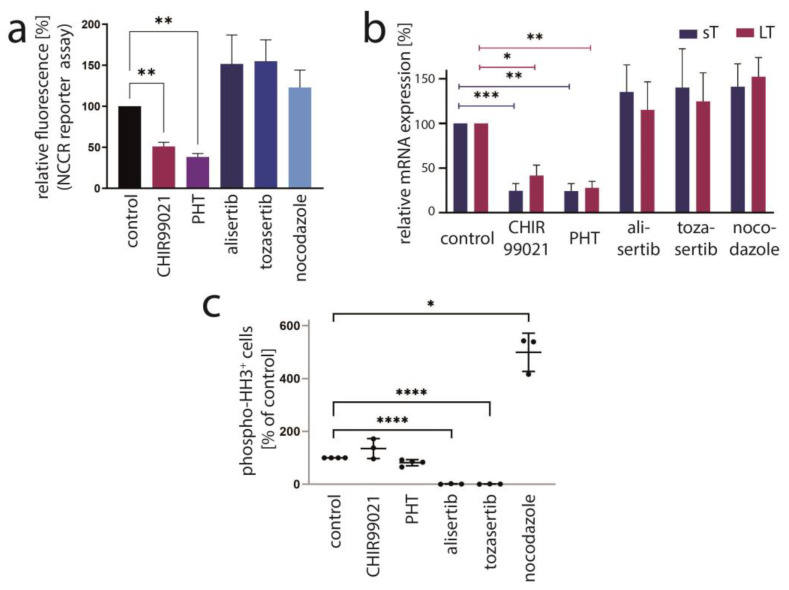
AURKA is not the crucial target mediating PHT-induced repression of TA expression. The effect of PHT (5 µM) treatment on MKL-1 cells was compared with application of other inhibitors including the AURKA inhibitor alisertib (5 µM), the AURKA/AURKB inhibitor tozasertib (500 nM), the spindle poison nocodazole (100 nM) and the GSK3 inhibitor CHIR99021 (10 µM). (**a**) Early region transcription was assessed by flow cytometry after a four-day treatment of MKL-1 cells carrying an NCCR reporter construct. Mean values (+SD) of the mean fluorescence intensities (normalized to controls) derived from three independent experiments are depicted. (**b**) TA mRNA expression after three hours of treatment was analyzed using real-time PCR with specific primers for MCPyV-LT and sT. *RPLP0* served as endogenous control for normalization. Mean values (+SEM) of six independent experiments are given. (**c**) To assess the inhibitory effect of the indicated drugs on AURKA in MKL1 cells, phosphorylation of histone H3 at serine 10 was analyzed by applying a fluorescence-labeled phospho-specific antibody and quantifying the percentage of positive cells by flow cytometry. Results from at least three independent experiments are displayed. Significance levels: *: ≤0.05; **: ≤0.01; ***: ≤0.001; ****: ≤0.0001.

PHT has been developed in an effort to identify compounds that specifically inhibit Aurora kinase A (AURKA). Indeed, it has been described to bear >1000-fold selectivity towards AURKA compared to AURKB [28]. Therefore, we next aimed to evaluate the role of AURKA in promoting TA expression in MCC cells. To this end, we first performed shRNA-induced knockdown of AURKA. However, although efficient repression of AURKA could be achieved by two different shRNAs, this did not translate into changes in LT mRNA or protein expression (Figure 3a,b). Combining PHT and *AURKA* shRNA followed by immunoblot analysis revealed that PHT repressed AURKA expression, although not as efficiently as the shRNA. Furthermore, this experiment demonstrated that PHT was still fully active in repressing TA when applied following shRNA induction despite its proposed target being already largely eliminated (Figure 3c). Since these results raised concerns whether the PHT effect on TA expression is mediated by targeting AURKA, we applied two alternative Aurora kinase inhibitors to MKL-1 cells: the AURKA-specific inhibitor alisertib [35] and tozasertib targeting AURKA and AURKB [36]. In contrast to PHT, both inhibitors induced a G2/M arrest (Supplementary Figure S2a,b), as described previously for Aurora kinase inhibition [37,38], questioning whether PHT targets this protein in MCC cells. Since a cell cycle arrest may result in profound secondary effects, we included nocodazole, a reversible inhibitor of microtubule polymerization, for comparison. Nocodazole also induced a G2/M arrest in MKL-1 cells very similar to alisertib and tozasertib (Supplementary Figure S2a,b). It has to be noted that PHT, in contrast to the significant reduction in S-phase cells observed after a 4-day treatment (Figure 1d), did not significantly block EdU incorporation after this 2-day treatment (Supplementary Figure S2b). Given the kinetics of LT reduction upon PHT treatment with only minor decreases after 1 and 2 days of treatment (Figure 1b) this diverging impact on cell cycle progression further supports the view that PHT inhibits cellular growth via repression of TA expression. With respect to the question of whether AURKA is involved in regulating TA expression in MCC cells, we next performed the NCCR reporter gene assay. While PHT repressed MCPyV early region transcription to a similar extent as the GSK3 inhibitor CHIR99021, none of alisertib, tozasertib or nocodazole reduced NCCR-driven expression (Figure 4a). Additionally, LT and sT mRNA levels are significantly reduced upon PHT or CHIR99021 treatment measured by real-time PCR after only 3 h of drug treatment (Figure 4b), suggesting a rather direct effect of both drugs on TA transcription. In contrast, the two Aurora kinase inhibitors alisertib and tozasertib did not induce such a reduction of sT or LT mRNA (Figure 4b). To evaluate the degree of AURKA inhibition by the different drugs, we analyzed phosphorylation of serine 10 of histone H3 (HH3). As expected, nocodazole treatment—arresting cells in mitosis—increased frequency of cells positive for phospho-HH3, while alisertib and tozasertib completely blocked HH3 phosphorylation (Figure 4c). The increase and inhibition were statistically significant, while the partial reduction in frequency of phospho-HH3 positive cells upon PHT treatment was not. This slight reduction might be due to AURKA inhibition by PHT but may also result from reduced cell cycling independent of AURKA inhibition.

In conclusion, several lines of evidence suggest that AURKA does not play a role in regulating MCPyV-NCCR-dependent transcription and that another protein might be the target of PHT crucial for regulating TA expression in MCC cells.

### 3.4. PHT Is a GSK3 Inhibitor

**Figure 5:**
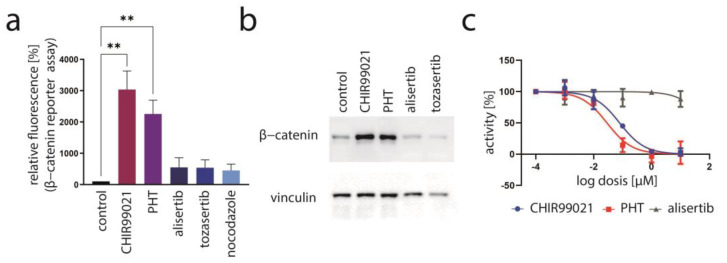
Evidence for PHT targeting GSK3. (**a**) Activation of the GSK3 target β-catenin was assessed by flow cytometry of MKL1 cells transduced with a β-catenin reporter construct following a four-day treatment. Depicted are mean values (+SD) of the mean fluorescence measured in five independent experiments. (**b**) Immunoblot analysis following four days of treatment with the indicated drugs. (**c**) The GSK3-inhibitory effect of the indicated drugs was evaluated using a commercial GSK3 kinase assay. Mean values of three independent measurements (±SD) are displayed. Significance level: **: ≤0.01.

Since most of the other kinase inhibitors identified to repress TA expression in our initial screen were targeting GSK3 [33], we speculated that PHT might also inhibit this kinase. Therefore, to evaluate whether PHT might bear GSK3-repressive activity, we used a reporter construct allowing us to monitor the activity of β-catenin, a transcription factor repressed by active GSK3 [39]. Indeed, PHT was found to induce β-catenin reporter fluorescence in the same order of magnitude as the potent GSK3 inhibitor CHIR99021, while alisertib and tozasertib had only minor and not statistically significant effects (Figure 3f). The effect of alisertib and tozasertib might be related to the G2/M arrest and subsequent cell death induced by these agents, since upon nocodazole treatment a similar slight increase was also observed (Figure 5a). A further indication that PHT is able to inhibit GSK3 in MKL-1 cells comes from immunoblot analysis. Indeed, the GSK3 target β-catenin, which is marked for proteasomal degradation by GSK3-dependent phosphorylation, accumulates following PHT as it does upon CHIR99021 treatment (Figure 5b). To formally prove that PHT might inhibit GSK3 kinase directly, we used a commercial GSK3b kinase kit. As expected, GSK3 kinase activity was clearly reduced by CHIR99021 (IC50: 79 nM), while alisertib had hardly any impact (Figure 5c). PHT, however, was also able to efficiently suppress GSK3 activity (IC50: 29 nM). In conclusion, these results indicate that PHT bears a so far unreported GSK3-inhibitory activity, and it is very likely that its ability to repress TA expression is related to targeting this kinase.

### 3.5. Inhibition of MCC Tumor Growth In Vivo by PHT

**Figure 6:**
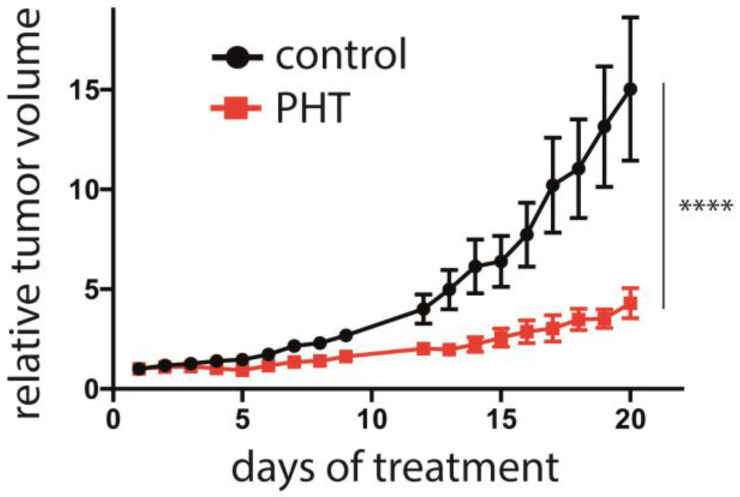
Repression of MCC tumor growth by PHT. MKL-1 cells embedded in Matrigel were injected subcutaneously into immunodeficient NOD/Scid mice. After the tumors had reached a size of approximately 150 mm^3^, the mice were randomly assigned to the control and the treatment group (*n* = 6 for PHT treatment and *n* = 5 for the control group, since in one animal no tumor growth was observed). PHT or 2% DMSO in PBS in the control animals were daily administered intraperitoneally and the tumor volume was determined. The experiment was terminated once individual tumors of the control group reached the maximum tolerable size. Depicted are the means (±SEM). Statistical analyses of the area under the curves revealed a significant difference (*p* < 0.0001; unpaired *t*-test). Significance level: ****: ≤0.0001.

Finally, the efficacy of PHT in vivo in was analyzed in an MCC xenotransplantation model [40]. After tumors were induced by subcutaneous injection of Matrigel-embedded MKL-1 cells in NOD/Scid mice, the mice were randomized into a control and a treatment group when tumor size was about 150 mm^3^. The control (2% DMSO) or PHT treatment were given by intraperitoneal injection. While the tumor grew inexorable in the mice of the control group, PHT treatment significantly impaired tumor growth (Figure 6). Of note, no abnormalities in the behavior of the mice were evident, nor did we observe any toxic side effects, suggesting that PHT treatment was well tolerated. Following termination of the experiment, the tumors were excised, and LT and TA expression were analyzed using immunohistochemistry and real-time PCR, respectively. However, this did not allow us to prove obvious differences in MCPyV sT/LT expression between control and PHT-treated tumors (Supplementary Figure S3). This might imply that tumor growth was restricted by a mechanism different from repression of TA expression. Alternatively, it is possible that the observed differences in tumor growth are due to a transient effect on TA expression and that in the course of the experiment, the effect of PHT has worn off. The latter is supported by the tumor growth characteristics: when normalized to day 17, the growth rate of the PHT-treated tumors during the last 3 days of the experiment was similar to the growth rate of untreated tumors (Supplementary Figure S4).

## 4. Discussion

The aim of this study was to characterize in detail a compound which in a cell-based assay has been identified as a substance inhibiting MCC proliferation by repressing TA expression and which had been described as an inhibitor of AURKA. AURKA is a serine/threonine kinase known to be crucially involved in various processes related to cell cycle progression, such as the G2/M transition, mitotic spindle assembly and DNA replication [41]. Moreover, it has been demonstrated that AURKA is a synthetic lethal partner of multiple tumor suppressors [42]. Therefore, development of specific AURKA inhibitors is thought to bear potential for cancer therapies. In such an effort, phthalazinone pyrazoles (including PHT used in this study) have been identified as potent inhibitors of Aurora kinase A with a 1000-fold increased selectivity compared to AURKB [28]. The authors also reported that PHT displayed phenotypic AURKA inhibition in the breast cancer cell line MCF7, with poorly formed centrosomes and subsequent arrest in G2/M. In contrast, we did not observe G2/M arrest induced by PHT in MKL-1 cells, suggesting that either AURKA suppression was not sufficient at the given concentration or that the MKL-1 cells are not sensitive to AURKA inhibition. The latter, however, is unlikely since we observed profound G2/M arrest with the AURKA inhibitor alisertib and the AURKA/AURKB inhibitor tozasertib. Interestingly, we are not the first to describe a blatant difference in the biological response towards PHT compared to other AURKA inhibitors. In this regard, out of a series of tested AURKA inhibitors, only PHT suppressed epithelial-mesenchymal transition during the differentiation of hepatocyte-like cells from human embryonic stem cells [43], suggesting that PHT might target different proteins depending on the cellular context. Indeed, these authors observed in their cells no reduction of AURKA autophosphorylation upon PHT treatment, while their data suggested that PHT might mediate its effect on the hepatocyte differentiation process by inhibiting AKT signaling. Amongst other things, this was evidenced by demonstration of reduced phosphorylation of GSK3β at serine9 [43] an established GSK3-inactivating AKT target site [44]. In contrast, we observed activation of β-catenin, a strong indication of GSK3 inactivation upon PHT treatment of MKL-1 cells. Additionally, we demonstrated that PHT can directly inhibit kinase activity of purified GSK3. Moreover, such a GSK3-inhibiting effect of PHT is a likely explanation for the observed repression of TA expression in MCC cells, since we recently demonstrated a role of GSK3 in promoting TA expression in these cancer cells [33]. Importantly, we demonstrate that this TA-repressing effect of PHT mediates a specific growth reduction of MCC cells, as it can be reverted by re-expression of TA. Moreover, PHT is capable of repressing MCC growth in vivo. However, the analyses of the tumors excised after termination of the experiment did not allow us to demonstrate that repression of TA expression is the mode of action of PHT slowing down MCC tumor growth in mice. Therefore, either another mechanism might be responsible for this effect, or the tumors have become resistant to PHT-induced TA repression in the course of the experiment. Indeed, it is a well-known phenomenon that tumors treated by small molecule inhibitors after an initial response can become resistant [45]. For example, tumors induced by xenotransplantation of a melanoma cell line first shrank upon treatment with the BRAF inhibitor vemurafenib, but after only 10 days regained growth [46]. Notably, growth characteristics of the PHT-treated tumors also suggest that they may have become PHT-resistant at the end of the experiment. Although rapid acquisition of resistance is not desired, an inhibitor—as the example of vemurafenib demonstrates—may still be clinically valuable. Hence, PHT might still be a potential therapeutic for MCPyV-positive MCC and might meet the needs of those patients with virus-related MCC refractory to anti-PD1/PD-L1 therapy [27].

Given that PHT acts as GSK3 inhibitor in MCC cells, it is worth noting that GSK3 inhibitors have been investigated as potential treatments for a variety of medical conditions, including Alzheimer’s disease [47], bipolar disorder [48], diabetes [49], inflammatory diseases [50] and cancer [51]. However, despite having shown promise in preclinical studies, their effectiveness in treating these diseases in humans still remains to be established, and the only approved drug affecting GSK3 is lithium chloride for the treatment of epilepsy and bipolar disorder [52]. Moreover, with respect to cancer, the role of GSK3 seems to be diverging. While in several cancer types, a tumor-suppressive role of GSK3—as negative regulator of the WNT signaling pathway—seems to be dominant, in others, a tumor-promoting function has been described [51]. In several clinical trials, GSK3 inhibitors are currently under investigation for the treatment of different solid cancers (e.g., colorectal and pancreatic cancer) (https://clinicaltrials.gov).

## 5. Conclusions

We have shown that 4-[(5-methyl-1H-pyrazol-3-yl)amino]-2H-phenyl-1-phthalazinone (PHT) represses growth of MCC cells in vitro specifically by inhibiting TA transcription. A limitation of our study is the fact that despite significant growth reduction of MCC xenografts in mice treated with PHT, we could not provide evidence that this is mediated by TA repression. Interestingly, although PHT has been developed as a specific inhibitor of AURKA, we demonstrate here that it also bears substantial activity suppressing GSK3. Importantly, we show that the TA-repressing effect in MCC is not related to inhibition of AURKA but most likely to PHT’s function as GSK3 inhibitor. 

## Data Availability

Data is contained within the article or Supplementary Material.

## Supplementary Materials

The following supporting information can be downloaded at: https://www.mdpi.com/article/10.3390/cancers15092542/s1. Figure S1: PHT treatment of MKL-1 cells and primary fibroblasts does not impact viability. Cells were incubated for three days with either 10 µM PHT or DMSO (1‰). Puromycin (2 µM) served as positive control for cell death induction. Following 72 hours of incubation, dead cells were identified by 7-AAD staining and flow cytometry analysis. Figure S2: No G2/M arrest induced by PHT in MKL-1 cells. The effect of PHT (5 µM) treatment on MKL-1 cells was compared with application of other inhibitors including the AURKA inhibitor alisertib (5 µM), the AURKA/AURKB inhibitor tozasertib (500 nM), the spindle poison nocodazole (100 nM) and the GSK3 inhibitor CHIR99021 (10 µM). Cell cycle analysis of MKL-1 cells treated for 48 h was performed by flow cytometry following EdU incorporation, fixation and Hoechst33342 staining. (a) Cell cycle profiles (only Hoechst 33342 fluorescence) of the treated cell populations are displayed. (b) Percentage of cells in the different cell cycle phases were determined in the EdU/Hoechst plots, and mean values (+SD) are given. Figure S3: No reduced expression of MCPyV-TA in xenograft MCC of PHT-treated mice after termination of the experiment. MKL-1 cells embedded in Matrigel were injected subcutaneously into immunodeficient NOD/Scid mice. After the tumors had reached a size of approximately 150 mm^3^, the mice were randomly assigned to the control and the treatment group (*n* = 6 for PHT treatment and *n* = 5 for the control group, since in one animal no tumor growth was observed). PHT or 2% DMSO in PBS in the control animals were daily administered intraperitoneally. The experiment was terminated once individual tumors of the control group reached the maximum tolerable size. The tumors were excised, RNA was isolated and TA mRNA expression was analyzed using real-time PCR and specific primers for MCPyV-LT and sT. RPLP0 served as endogenous control for normalization. The values for individual tumors normalized to the lowest value in the control group are given. Figure S4: Similar growth rates of control and PHT-treated tumors during the last three days of the mouse experiment (corresponding to Figure 6 in the publication). MKL-1 cells embedded in Matrigel were injected subcutaneously into immunodeficient NOD/Scid mice. After the tumors had reached a size of approximately 150 mm^3^, the mice were randomly assigned to the control and the treatment group (*n* = 6 for PHT treatment and *n* = 5 for the control group, since in one animal no tumor growth was observed). PHT or 2% DMSO in PBS in the control animals were daily administered intraperitoneally, and the tumor volume was determined. After 20 days of treatment, the experiment was terminated. Here, only the tumor mean volumes (±SD) of the last three days normalized to the volumes of day 17 are depicted.
