# Supplementary material for: 4-[(5-Methyl-1H-pyrazol-3-yl)amino]-2H-phenyl-1-phthalazinone Inhibits MCPyV T Antigen Expression in Merkel Cell Carcinoma Independent of Aurora Kinase A
Source: Cancers (Basel). 2023 Apr 28;15(9):2542. doi: 10.3390/cancers15092542 (PMC10177447; doi:10.3390/cancers15092542)
Supplement: Supplementary file 1 [file cancers-15-02542-s001.zip › cancers-2266663-supplementary.pdf]

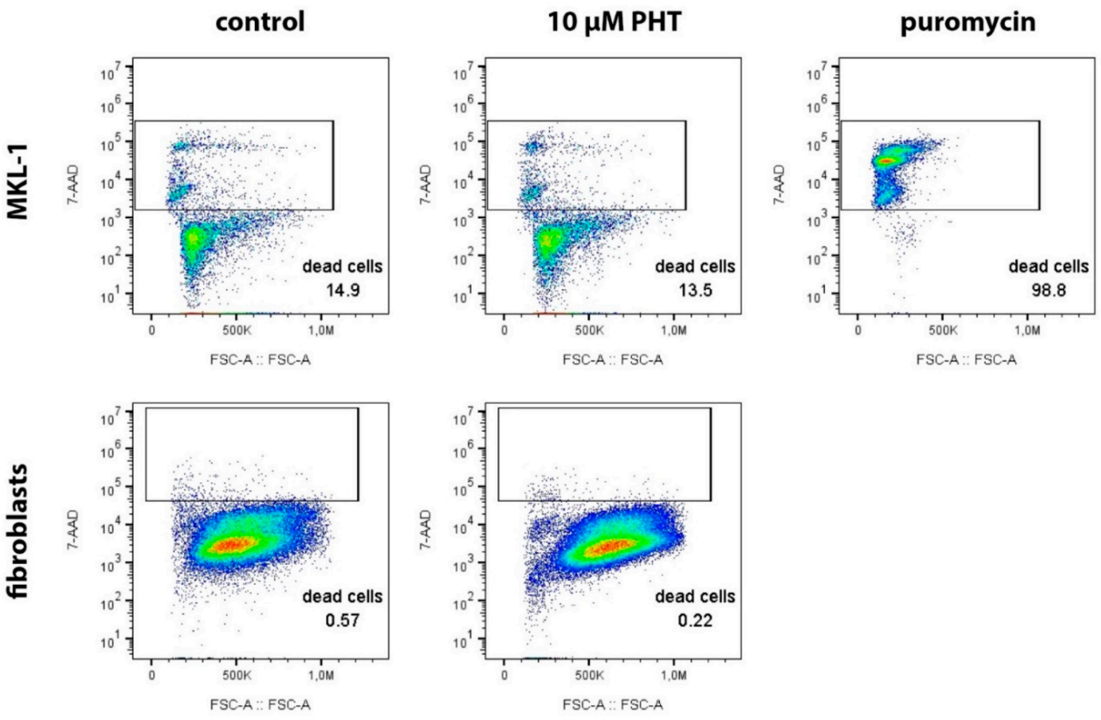

Figure S1. PHT treatment of MKL-1 cells and primary fibroblasts does not impact viability. Cells were incubated for three days with either 10 µM PHT or DMSO (1‰). Puromycin (2 µM) served as positive control for cell death induction. Following 72 hours of incubation, dead cells were identified by 7-AAD staining and flow cytometry analysis.

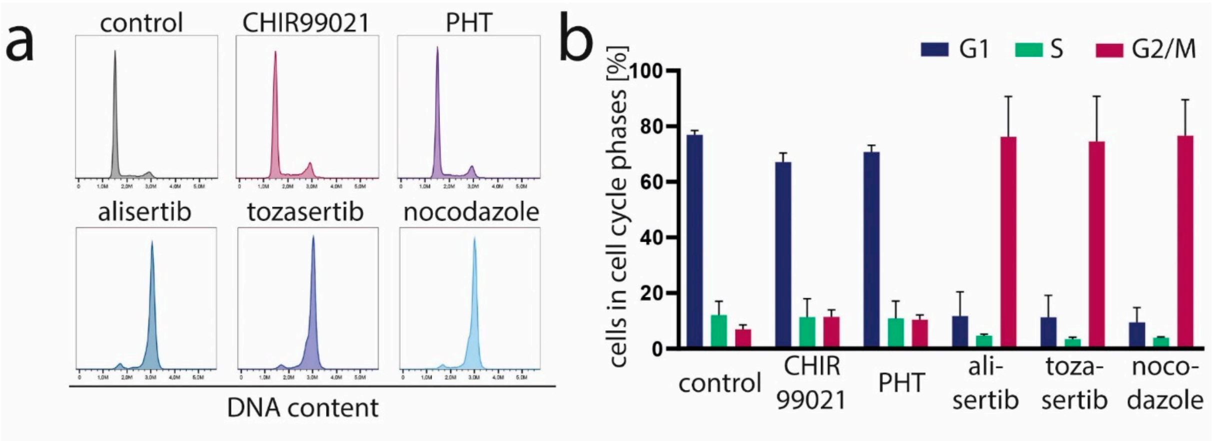

Figure S2. No G2/M arrest induced by PHT in MKL-1 cells. The effect of PHT (5 µM) treatment on MKL-1 cells was compared with application of other inhibitors including the AURKA inhibitor alisertib (5 µM), the AURKA/AURKB inhibitor tozasertib (500 nM), the spindle poison nocodazole (100 nM) and the GSK3 inhibitor CHIR99021 (10 µM). Cell cycle analysis of MKL-1 cells treated for 48 h was performed by flow cytometry following EdU incorporation, fixation and Hoechst33342 staining. (a) Cell cycle profiles (only Hoechst 33342 fluorescence) of the treated cell populations are displayed. (b) Percentage of cells in the different cell cycle phases were determined in the EdU/Hoechst plots, and mean values (+SD) are given.

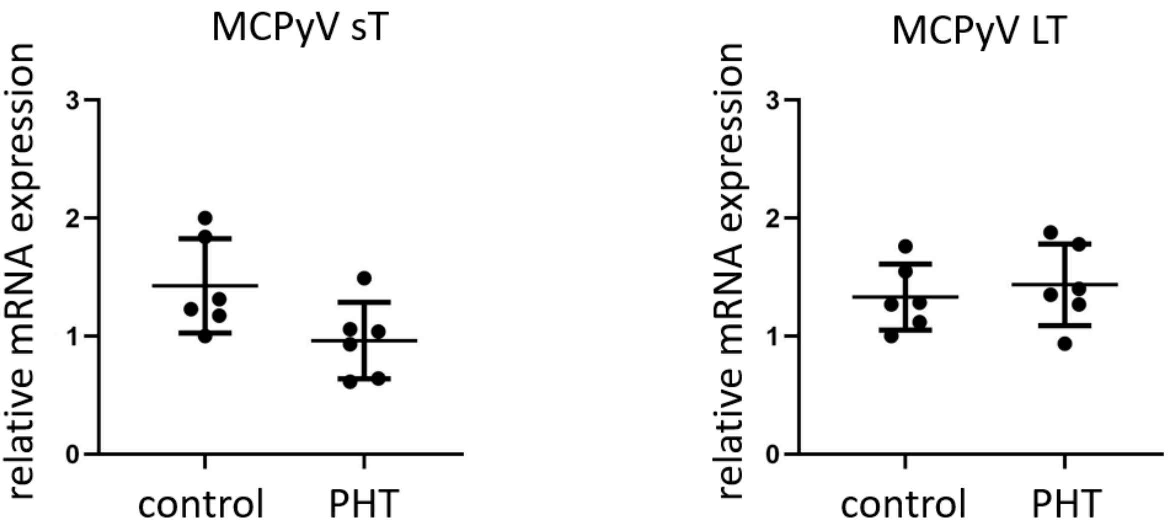

Figure S3. No reduced expression of MCPyV-TA in xenograft MCC of PHT-treated mice after termination of the experiment. MKL-1 cells embedded in Matrigel were injected subcutaneously into immunodeficient NOD/Scid mice. After the tumors had reached a size of approximately 150 mm^3, the mice were randomly assigned to the control and the treatment group ($n = 6$ for PHT treatment and $n = 5$ for the control group, since in one animal no tumor growth was observed). PHT or 2% DMSO in PBS in the control animals were daily administered intraperitoneally. The experiment was terminated once individual tumors of the control group reached the maximum tolerable size. The tumors were excised, RNA was isolated and TA mRNA expression was analyzed using real-time PCR and specific primers for MCPyV-LT and sT. RPLP0 served as endogenous control for normalization. The values for individual tumors normalized to the lowest value in the control group are given.

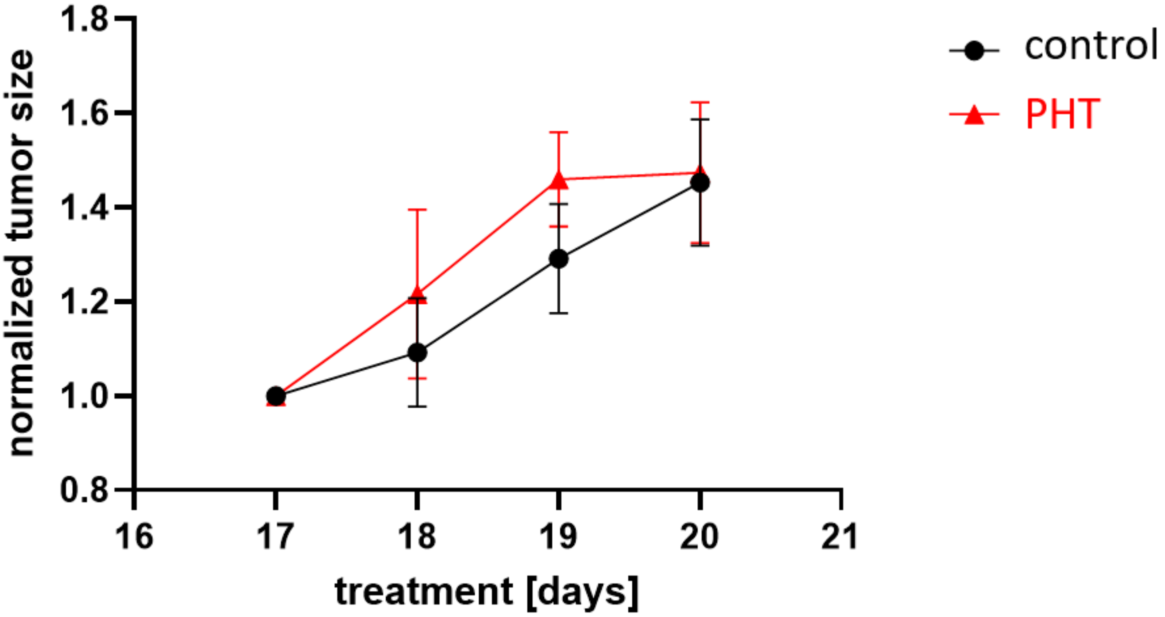

Figure S4. Similar growth rates of control and PHT-treated tumors during the last three days of the mouse experiment (corresponding to Figure 6 in the publication). MKL-1 cells embedded in Matrigel were injected subcutaneously into immunodeficient NOD/Scid mice. After the tumors had reached a size of approximately 150 mm^3, the mice were randomly assigned to the control and the treatment group ($n = 6$ for PHT treatment and $n = 5$ for the control group, since in one animal no tumor growth was observed). PHT or 2% DMSO in PBS in the control animals were daily administered intraperitoneally, and the tumor volume was determined. After 20 days of treatment, the experiment was terminated. Here, only the tumor mean volumes (±SD) of the last three days normalized to the volumes of day 17 are depicted.
